# Spontaneous Resolution of a Unilateral Epiretinal Membrane in Bilateral Peripheral Retinal Vasculopathy: A Case Report

**DOI:** 10.3390/reports9040358

**Published:** 2026-10-16

**Authors:** Alessandro Avitabile, Maria Ludovica Ruggeri, Alberto Quarta, Corina Valentina de Santis Ciacci, Vincenza Maria Elena Bonfiglio

**Affiliations:** 1 Biomedicine, Neuroscience and Advance Diagnostic (BIND) Department, University of Palermo, 90133 Palermo, Italy; alessandro.avitabile@unipa.it; 2 Eye Clinic, University Hospital of Chieti-Pescara, 66100 Chieti, Italy; 3 Department of Neuroscience, Imaging and Clinical Science, “G. d’Annunzio” University Chieti-Pescara, 66100 Chieti, Italy

**Keywords:** epiretinal membrane, spontaneous resolution, peripheral retinal vasculopathy, vitritis, case report

## Abstract

**Background and Clinical Significance**: Epiretinal membranes (ERMs) are uncommon in young patients, and spontaneous anatomical resolution is rare. We report spontaneous disappearance of a unilateral ERM after marked worsening in an 18-year-old woman with bilateral peripheral occlusive/telangiectatic retinal vasculopathy and vitritis of undetermined etiology. **Case Presentation**: She presented with right-eye (RE) metamorphopsia and uncorrected visual acuity of 20/25. Optical coherence tomography (OCT) showed a macular ERM; fluorescein angiography showed bilateral peripheral telangiectasia, leakage, vascular truncation, and capillary nonperfusion. Bilateral vitritis was present. Treatment comprised a single intravitreal ranibizumab injection in the RE and peripheral laser photocoagulation in both eyes for the vascular abnormalities; the ERM was managed conservatively. Before its disappearance, the ERM showed marked anatomical worsening accompanied by progressive visual decline. RE central macular thickness (CMT) peaked at 901 µm on Day 71; the lowest uncorrected visual acuity, 20/200, was recorded on Days 166 and 187. Posterior vitreous detachment was not clinically detectable throughout follow-up. On Day 243, the ERM was first documented as absent clinically and on OCT; visual acuity with correction was 20/30, CMT was 464 µm, and vitritis was no longer clinically detectable. At approximately 38 months, macular contour remained restored and recorded visual acuity was 20/20 in both eyes. When mapped to the MANE Select transcript, the retained VCAN variant notation corresponds to NM_004385.5:c.2234A>C (p.Lys745Thr); ClinVar reports conflicting classifications, and the finding was therefore considered non-diagnostic. **Conclusions**: This case documents spontaneous ERM disappearance without vitreoretinal surgery after marked anatomical and functional worsening. The separation mechanism and any causal contribution of ranibizumab or laser photocoagulation remain uncertain.

## 1. Introduction and Clinical Significance

An epiretinal membrane (ERM) is a fibrocellular proliferation on the inner retinal surface that may cause macular distortion, metamorphopsia, and reduced visual acuity [1]. ERM prevalence increases with age, and the condition is rare in children and young adults [1,2]. In pediatric patients, reported associations include ocular trauma, uveitis, and other ocular disorders, although idiopathic cases also occur [2].

Spontaneous ERM separation has been described, particularly in younger patients, although it remains uncommon [3,4,5]. In a series of 1248 eyes with idiopathic ERM, spontaneous separation occurred in 37 eyes (3.0%) [4]. Proposed mechanisms include posterior vitreous detachment (PVD), tangential membrane contraction, membrane rupture, and extracellular-matrix remodeling [3,4,5]. In many reported cases, the exact mechanism remains uncertain because the separation event and posterior hyaloid status are not documented by serial optical coherence tomography.

We report the spontaneous disappearance of a unilateral ERM after marked anatomical and functional worsening in an 18-year-old patient with bilateral peripheral occlusive/telangiectatic retinal vasculopathy. The primary aim of this report is to document the longitudinal clinical and multimodal imaging course of the ERM and to discuss possible mechanisms of its disappearance. The vascular phenotype and its treatment are presented as clinical context, and their relationship to the ERM course remains uncertain.

## 2. Case Presentation

**Figure 1:**
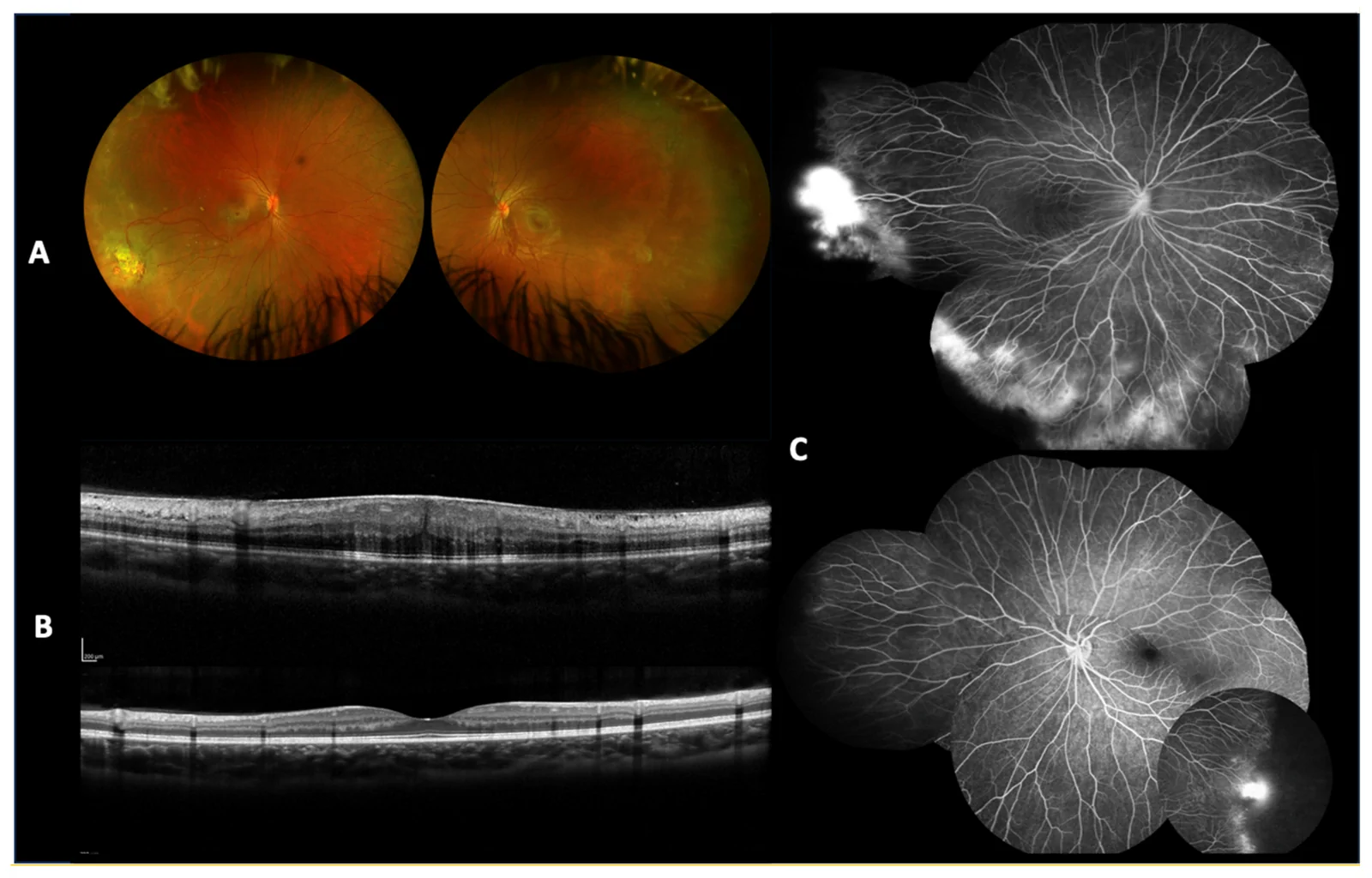
Baseline multimodal imaging. (**A**) Color fundus photographs of the right and left eyes. (**B**) Macular optical coherence tomography (OCT) B-scans showing an epiretinal membrane (ERM) in the right eye and an unremarkable macular contour in the left eye. (**C**) Available wide-field fluorescein angiography (FA) frames showing bilateral peripheral vascular abnormalities, including leakage and nonperfusion. The available figure contains one selected angiographic frame per eye; phase timing and enlarged macular/optic-disc views were not available.

An 18-year-old woman presented on Day 0 with reduced visual acuity and metamorphopsia in her right eye (RE). She reported no known systemic or ocular disease, history of ocular surgery, or relevant family history. Baseline visual acuity, measured without correction, was 20/25 in the RE and 20/20 in the left eye (LE); the Amsler test was positive in the RE and negative in the LE. Intraocular pressure was 17 mmHg in both eyes by Goldmann applanation tonometry. The corneas and lenses were clear. Dilated fundus examination showed a macular epiretinal membrane (ERM) in the RE, together with dilated telangiectatic vessels and other vascular abnormalities in the temporal midperiphery (Figure 1). PVD was not clinically detectable in either eye at presentation or during follow-up. Bilateral vitritis was also present, more pronounced in the RE, and was characterized by vitreous cells and opacities. Formal inflammatory grading was not documented. Axial length was unavailable. Macular optical coherence tomography (OCT) confirmed the ERM, and central macular thickness (CMT) in the RE was 568 µm on Day 1. Fluorescein angiography (FA) performed on Day 1 demonstrated bilateral peripheral telangiectatic and tortuous vessels, vascular leakage, areas of capillary nonperfusion, and abrupt peripheral vascular termination, most prominently in the temporal midperiphery. No optic-disc leakage was observed in either eye. In the RE, FA additionally showed progressive macular hyperfluorescence and tractional distortion of the macular vessels consistent with ERM-related traction. Indocyanine green angiography showed hypercyanescence corresponding to an abnormal vascular network in the inferotemporal periphery, considered suggestive of neovascularization. Taken together, the clinical and multimodal imaging findings supported a descriptive diagnosis of bilateral peripheral occlusive/telangiectatic retinal vasculopathy of undetermined etiology, with a coexisting ERM in the RE.

The treatment strategy focused on the angiographically active peripheral vascular abnormalities, with conservative management of the ERM. On Day 2, a single intravitreal injection of ranibizumab was administered to the RE to reduce leakage and activity associated with the suspected peripheral vascular proliferation. On Day 20, argon laser photocoagulation was subsequently applied to areas of peripheral capillary nonperfusion over 360° in the RE and 180° in the LE.

A targeted systemic work-up, including complete blood count, inflammatory markers, urinalysis, syphilis and tuberculosis screening, HIV testing, ANA, anti-dsDNA antibodies, complement levels, ANCA, and antiphospholipid antibodies, was unrevealing. Genetic testing was reported as negative for familial exudative vitreoretinopathy (FEVR)-associated genes. The original laboratory report, tested gene list, analytical methods, coverage metrics, and information on deletion/duplication analysis were unavailable for review. The scope of the reported negative result could therefore not be independently assessed. The only retained variant notation was VCAN c.2234A>C. When referenced to the MANE Select transcript NM_004385.5, this corresponds to p.(Lys745Thr). ClinVar currently reports conflicting germline classifications—uncertain significance, benign, and likely benign conflicting classifications [6]; the finding was therefore considered non-diagnostic. The same VCAN variant had been reported in a parent described as asymptomatic. Detailed ophthalmic examination findings and retinal imaging of relatives were unavailable.

**Figure 2:**
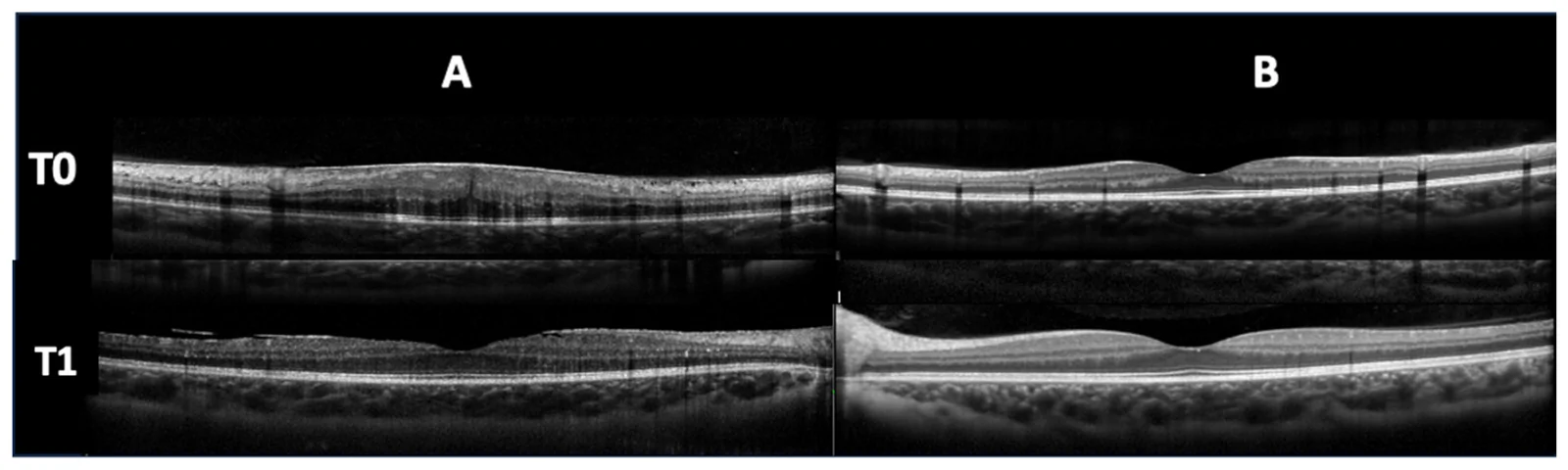
Available OCT images at baseline and two-year follow-up. Macular OCT B-scans of the right (**A**) and left eye (**B**) at baseline (T0) and two years (T1). The epiretinal membrane (ERM) visible in the right eye at baseline is no longer visible at T1.

On Day 26, uncorrected visual acuity had decreased to 20/100 RE and 20/25 LE, while central macular thickness in the RE had increased to 724 µm. On Day 71, follow-up FA documented residual peripheral nonperfusion in both eyes, and RE CMT reached its highest recorded value of 901 µm. Additional bilateral laser photocoagulation was performed on Day 113, with 1800 spots delivered to the RE and 1000 spots to the LE. Between Days 166 and 215, RE CMT remained markedly elevated but essentially stable at approximately 850 µm. The lowest recorded uncorrected visual acuity in the RE was 20/200 on Days 166 and 187. On Day 243, RE visual acuity had improved to 20/30 with −0.75 D sphere and −0.50 D cylinder × 180°, and the ERM was no longer visible clinically or on OCT; the previously observed vitreous cells and opacities were no longer clinically detectable in either eye. Small intraretinal hyperreflective foci were noted, and CMT measured 464 µm. By Day 488, RE CMT had further decreased to 327 µm, with best-corrected visual acuity (BCVA) improving to approximately 20/25 in the RE and 20/20 in the LE. At the final follow-up, approximately 38 months after presentation, the restored macular contour was maintained, recorded visual acuity was 20/20 in both eyes, and the hyperreflective foci were reduced. No vitrectomy or membrane peeling was performed. The clinical course is summarized in Table 1. Available baseline and two-year OCT images are shown in Figure 2.

## 3. Discussion

Spontaneous ERM resolution is uncommon, particularly after an interval of anatomical and functional worsening. In the present case, the membrane disappeared during follow-up in an 18-year-old patient with bilateral peripheral occlusive/telangiectatic retinal vascular abnormalities and vitritis treated with intravitreal ranibizumab and peripheral laser photocoagulation. The temporal sequence raises questions regarding the mechanism of ERM separation and whether the associated vitritis and vascular phenotype or its treatment influenced the course of the membrane.

Spontaneous ERM separation has been described in young patients. Meyer et al. reported six patients aged 15–30 years in whom sequential OCT showed progressive membrane peeling and foveal normalization [3]. In a series of 1248 eyes with idiopathic ERM, Yang et al. observed self-separation in 37 eyes (3.0%) and identified different OCT patterns according to the presence or absence of pre-existing PVD [4]. Proposed mechanisms include PVD-mediated peeling, tangential contraction exceeding retinal adhesion, membrane rupture, and extracellular-matrix remodeling [3,4,5]. In our patient, PVD was not clinically detectable at presentation or during follow-up, so a PVD-mediated mechanism is not supported by the clinical examination findings. Vitritis was no longer clinically detectable at the visit when ERM disappearance was first documented. This observation raises the possibility that subsiding inflammatory activity and associated glial remodeling contributed to membrane separation, as proposed for inflammation-associated ERMs [5]. This hypothesis remains speculative because the separation event was not captured on serial OCT.

The intravitreal anti-VEGF injection and laser photocoagulation were directed at peripheral leakage, suspected vascular proliferation, and capillary nonperfusion rather than at the ERM. The subsequent macular course was biphasic: an early decline in visual acuity accompanied by increased CMT was followed, over longer follow-up, by disappearance of the ERM, restoration of the macular contour, and recovery of visual acuity. Comparable sequences have been described in secondary ERMs associated with retinal vascular disorders. Ding et al. reported spontaneous release of epiretinal membrane after photocoagulation in seven of eight eyes with ERMs secondary to retinal vascular tumors; among the six uncomplicated cases managed without vitreoretinal surgery, macular anatomy normalized, with BCVA improving in five eyes and remaining stable in one [7]. The broader literature also includes ERM release in association with treated Coats disease, Leber’s miliary aneurysms, and other vascular retinal lesions, suggesting that changes in vascular leakage, retinal edema, gliotic contraction, or the balance of tractional forces may influence the behavior of a secondary ERM [5]. These clinical settings are not directly equivalent to the bilateral peripheral occlusive/telangiectatic vasculopathy observed in our patient, and the use of both anti-VEGF therapy and laser photocoagulation during the same treatment period precludes attribution of the subsequent ERM resolution to either intervention.

The vascular phenotype remained etiologically indeterminate. Bilateral vitritis, vascular leakage, and capillary nonperfusion were compatible with an inflammatory retinal vascular process, but the absence of optic-disc leakage and the unrevealing systemic work-up did not establish a specific inflammatory etiology; isolated ocular inflammation therefore remained possible [8]. Peripheral telangiectasia and leakage also overlapped with Coats disease. Although subtle vascular abnormalities have been reported in fellow eyes, classic Coats disease remains predominantly unilateral and exudative; the bilateral leakage and nonperfusion observed in our patient, without marked lipid exudation or exudative retinal detachment, were therefore less typical of this diagnosis [9,10]. Among the entities considered, the closest phenotypic overlap was with FEVR, given the bilateral peripheral nonperfusion, vascular truncation, telangiectatic terminal vessels, and leakage [11]. Nevertheless, the available familial and genetic findings did not establish FEVR, and the angiographic findings were therefore interpreted as an FEVR-like pattern rather than definitive evidence of an inherited disorder. Accordingly, bilateral peripheral occlusive/telangiectatic retinal vasculopathy of undetermined etiology remained the most appropriate diagnostic framework.

The VCAN finding was considered non-diagnostic. ClinVar reports conflicting classifications for NM_004385.5:c.2234A>C (p.Lys745Thr)—uncertain significance, benign, and likely benign [6]. The absence of the characteristic vitreous phenotype of VCAN-related vitreoretinopathy, together with identification of the same variant in an asymptomatic parent, did not support a diagnosis of Wagner syndrome or a causal relationship with the peripheral vascular abnormalities [12,13].

Small intraretinal hyperreflective foci were noted when ERM disappearance was first documented and subsequently decreased. On the available OCT images, they were distributed predominantly within the middle retinal layers, approximately at the level of the inner nuclear layer (INL) and adjacent plexiform layers. This topography places them close to the intermediate and deep capillary plexuses that border the INL [14]. In other vascular retinopathies, similarly located foci have been observed in close relation to retinal capillaries and may represent perivascular deposition of extravasated macromolecules or lipid-laden macrophages, while activated microglial cells remain another possible cellular substrate [15,16]. Accordingly, in this case, the foci may reflect ischemic or exudative changes in the setting of the vascular phenotype, a microglial/macrophage-related cellular response, or nonspecific post-treatment remodeling; their presence and subsequent reduction do not provide independent evidence of ocular inflammation.

The main limitations of this report are the retrospective single-case design and the absence of a definitive etiologic diagnosis for the bilateral vascular phenotype. In addition, the coexistence of vasculopathy, vitritis, anti-VEGF exposure, and staged laser treatment prevents separation of their potential contributions to ERM remodeling. These limitations preclude mechanistic or treatment-causality claims but do not alter the directly observed longitudinal finding of ERM disappearance without vitreoretinal surgery.

Complete time-stamped early and late FA phases, enlarged macular and optic-disc views, and serial OCT scans immediately bracketing ERM disappearance were unavailable; consequently, the posterior hyaloid status, ERM-edge separation, and exact separation event could not be documented.

Complete longitudinal refractive data, axial length, standardized inflammatory grading, and detailed ophthalmic imaging of relatives were also unavailable.

Future prospective cohorts including resolving and persistent ERMs in patients with comparable vascular phenotypes could help identify features associated with spontaneous release. Serial OCT assessment of posterior hyaloid status and ERM attachment, together with standardized documentation of inflammatory and vascular activity and treatment timing, would help characterize the sequence of changes preceding release.

## 4. Conclusions

This case highlights an unusual course of spontaneous anatomical resolution of a unilateral ERM, with restoration of macular architecture and recovery of visual acuity after a period of marked worsening, in an 18-year-old patient with bilateral peripheral occlusive/telangiectatic retinal vasculopathy and vitritis of undetermined etiology. The disappearance of the membrane without vitreoretinal surgery illustrates that favorable spontaneous evolution may occur in young patients even in the setting of complex peripheral retinal vascular abnormalities. Although the precise mechanism of resolution and any contribution of anti-VEGF therapy or laser photocoagulation cannot be determined from this observation, the longitudinal clinical and imaging course remains noteworthy. The associated VCAN finding should be interpreted only in conjunction with complete variant classification and concordant clinical and familial evidence. Serial multimodal imaging, including OCT, may support careful observation in selected young patients when immediate vitreoretinal surgery is not otherwise required.

## Figures and Tables

**Table 1:** Clinical timeline, visual acuity, imaging findings, and treatment.

Relative Time	Recorded Visual Acuity, RE/LE (Snellen)	CMT RE, µm	Principal Finding or Intervention
Day 0–1	20/25; 20/20 (uncorrected)	568	Symptomatic macular ERM RE; no clinically detectable PVD in either eye, bilateral vitritis RE > LE. FA demonstrated bilateral peripheral telangiectatic vessels, leakage, vascular truncation, and capillary nonperfusion.
Day 2	—	—	Single intravitreal ranibizumab injection RE
Day 20	—	—	Initial argon laser photocoagulation in both eyes
Day 26	20/100; 20/25 (uncorrected)	724	Increased macular thickness RE
Day 71	—	901	Repeat FA demonstrated residual peripheral nonperfusion; highest recorded CMT
Day 113	—	—	Additional laser photocoagulation: 1800 spots RE and 1000 spots LE
Day 166	20/200; 20/25 (uncorrected)	857	Lowest recorded visual acuity RE
Day 243	20/30; 20/20 (−0.75 D sphere; −0.50 D cylinder × 180°)	464	ERM first documented as absent clinically and on OCT; small intraretinal hyperreflective foci noted. Vitritis was no longer clinically detectable in both eyes
Day 488	20/25; 20/20	327	Further reduction in macular thickness with continued visual recovery

Note: RE, right eye; LE, left eye; CMT, central macular thickness; ERM, epiretinal membrane; PVD, posterior vitreous detachment; FA, fluorescein angiography; OCT, optical coherence tomography.

## Data Availability

Data are unavailable due to privacy or ethical restrictions.
